# Chemotherapeutic treatment efficacy and sensitivity are increased by adjuvant alternating electric fields (TTFields)

**DOI:** 10.1186/1756-6649-9-1

**Published:** 2009-01-08

**Authors:** Eilon D Kirson, Rosa S Schneiderman, Vladimír Dbalý, František Tovaryš, Josef Vymazal, Aviran Itzhaki, Daniel Mordechovich, Zoya Gurvich, Esther Shmueli, Dorit Goldsher, Yoram Wasserman, Yoram Palti

**Affiliations:** 1 NovoCure Ltd., MATAM Advanced Technology Centre, Haifa 31905, Israel; 2 Na Homolce Hospital, Roentgenova 2, Prague 5, 150 30, Czech Republic; 3 Rambam Medical Center, PO Box 9602, Haifa 31096, Israel; 4 B. Rappaport Faculty of Medicine, Technion – Israel Institute of Technology, Technion City, Haifa 32000, Israel

## Abstract

**Background:**

The present study explores the efficacy and toxicity of combining a new, non-toxic, cancer treatment modality, termed Tumor Treating Fields (TTFields), with chemotherapeutic treatment in-vitro, in-vivo and in a pilot clinical trial.

**Methods:**

Cell proliferation in culture was studied in human breast carcinoma (MDA-MB-231) and human glioma (U-118) cell lines, exposed to TTFields, paclitaxel, doxorubicin, cyclophosphamide and dacarbazine (DTIC) separately and in combinations. In addition, we studied the effects of combining chemotherapy with TTFields in an animal tumor model and in a pilot clinical trial in recurrent and newly diagnosed GBM patients.

**Results:**

The efficacy of TTFields-chemotherapy combination in-vitro was found to be additive with a tendency towards synergism for all drugs and cell lines tested (combination index ≤ 1). The sensitivity to chemotherapeutic treatment was increased by 1–3 orders of magnitude by adjuvant TTFields therapy (dose reduction indexes 23 – 1316). Similar findings were seen in an animal tumor model. Finally, 20 GBM patients were treated with TTFields for a median duration of 1 year. No TTFields related systemic toxicity was observed in any of these patients, nor was an increase in Temozolomide toxicity seen in patients receiving combined treatment. In newly diagnosed GBM patients, combining TTFields with Temozolomide treatment led to a progression free survival of 155 weeks and overall survival of 39+ months.

**Conclusion:**

These results indicate that combining chemotherapeutic cancer treatment with TTFields may increase chemotherapeutic efficacy and sensitivity without increasing treatment related toxicity.

## Background

A new physical cancer treatment modality termed Tumor Treating Fields, or TTFields, has recently been demonstrated to be highly effective when applied to cell cultures, animal cancer models, as well as to patients suffering from locally advanced and or metastatic solid tumors [1-3]. In a pilot clinical trial, the medians of time to disease progression and overall survival of recurrent GBM patients treated by TTFields alone were more than double the reported medians of historical control patients [1]. In contrast to the widely used physical treatment modality, ionizing radiation, TTFields are not associated with significant side effects.

TTFields are low intensity (1–2 V/cm), intermediate frequency (100 – 200 kHz) alternating electric fields generated by special insulated electrodes applied to the skin surface. These specially tuned fields have no effect on quiescent cells while having an anti-mitotic effect on dividing cells. During cytokinesis, TTFields generate non-uniform intracellular fields that exert forces that move polar macromolecules and organelles towards the narrow neck, separating the newly forming daughter cells, by a process termed dielectrophoresis. These molecular and organelle movements, together with an interference with the spindle tubulin polymerization process, inhibit cell division and lead to cell death[2]. Fortunately, the dividing cells of the hematopoietic system are not affected by TTFields as the muscles surrounding the marrow containing bones serve as an effective electric field shield. Moreover, due to their relatively high frequency range and very low intensity, TTFields do not stimulate nerves and muscles, do not generate meaningful temperature elevation or puncture the cell membrane (as the strong electroporation fields do [4]). Thus, TTFields are not associated with meaningful toxicity in contrast to most anti-cancer agents currently in use [5].

In view of the unfavorable therapeutic indexes of the available effective chemical and physical (i.e. ionizing radiation) therapeutic agents, many cancer treatment protocols require simultaneous or sequential use of a number of therapeutic agents in an attempt to increase efficacy while maintaining tolerable toxicity [5-7]. Within this framework it is generally accepted that by adding ionizing radiation [8] to chemotherapy one gets both the benefit of the radiation effect as well as sensitization leading to an increased efficacy without a corresponding increase in toxicity. On the basis of the above this study explores the potential use of the new physical treatment modality, TTFields, in combination with chemotherapeutic agents in cell cultures, an animal tumor model, as well as in patients with glioblastoma (GBM). As TTFields are not associated with systemic toxicity [1] the expectation is that their addition will result in an increase in efficacy alone.

## Methods

### Cell cultures

Cells were cultured and maintained as previously described [1,2]. In brief: Human breast cancer (MDA-MB-231) and human glioma (U-118) obtained from ATCC (USA) were cultured in DMEM + 10% FCS media in a 5% CO_2 _incubator at 37°C. Drops consisting of 200 μl suspension of cells (100 × 10^3 ^cells/ml) were placed at the centre of 35 mm Petri dishes, incubated for 2 hours to allow for cell attachment, then 1.5 ml of media were added and incubation was continued for an additional 22 h. Following this, the baseline cell count was estimated using the XTT colorimetric method (expressed as OD_0_). The media in the Petri dishes was replaced by fresh media (3 ml), with or without a chemotherapeutic agent and incubated at a final temperature of 37° ± 0.5°C for 24 to 72 hours after which the cell number was re-estimated (OD_1_). The relative number of viable cells at each time point following baseline was expressed as OD_1_/OD_0 _and treatment efficacy as the % change in proliferation relative to control:

(1) (OD_1_/OD_0_)_experiment _* 100/(OD_1_/OD_0_)_control_

### TTFields treatment of cultures

As previously described [1,2], two pairs of electrodes, insulated by a high dielectric constant ceramic, were positioned normal to each other at a distance of 20 mm in treatment and control dishes. In the former, the electrodes were connected to sinusoidal waveform generator that generated fields of optimal frequencies in the medium [1,2,9]: 150 kHz for breast cancer and 200 kHz for glioma, that changed direction by 90° every 250 ms. Field intensity was measured as described previously [2] and expressed as V/cm. For 72 h experiments the TTFields intensity of 1.75 V/cm was used. For 24 h experiments 0.65, 1.25 and 1.75 V/cm TTFields were used.

Four different sets of experiments were conducted in conjunction with each chemotherapeutic agent: untreated sham control, treatment with TTFields, treatment with the chemotherapeutic agents, and combined TTFields – Chemo treatment.

### Assessment of combination Index and dose reduction index

The Chou and Talalay [10] method for assessing the combined effect of multiple drugs was used for the drug – TTFields combinations. In order to assess whether the interactions between TTFields and each of the chemotherapeutic agents is synergistic, additive or antagonistic, combination indexes were calculated as follows; TTFields intensity replaced the concentration (dose) variable in the analyses. Dose-response curves were generated for TTFields and each drug to determine the median effect points. Variable ratios of drug concentrations to TTFields intensities were used to calculate the Combination Indexes (CI) as follows:

(2) CI = (C_Drug(incombination), X% effect_/C_Drug(alone), X% effect_) + (I_TTFields(incombination), x% effect _/I_TTFields(alone), X% effect_)

Where: C are the drug concentrations and I the TTFields intensities use to achieve a preset X% effect. Relationships of CI<1 indicate more than additive – synergy, CI = 1 reflects additivity – summation and CI>1 indicates less than additive or antagonism.

In order to asses whether TTFields increase the sensitivity of tumor cells to various chemotherapeutic agents, the dose reduction index (DRI) of for each of these agents was calculated according to [11]. In short, the median-effect plots were for each chemotherapy-TTFields combination, were constructed. The ratio of affected to unaffected number of cells (f_a_/f_u_) was plotted versus drug concentration on a log-log scale. The median effect point (D_m_) was assessed by deriving the slope of the linear regression for each of the plots. The DRI for a 50% effect (DRI_m_) was calculated as the ratio of D_m _for drug alone and for combined drug-TTFields:

(3) DRI_m _= D_m(drugalone)_/D_m(combinedtreatment)_

A DRI greater than 1 indicates an increase in sensitivity to the drug. The greater the DRI, the more significant the possible dose reduction.

### In-vivo experiments

Combined TTFields and Paclitaxel efficacy study in VX2 tumor bearing rabbits was conducted after approval by the NovoCure Internal Animal Care and Use Committee. All painful or anxiogenic procedures were performed under general anesthesia induced by intramuscular administration of 30 mg/kg of ketamine hydrochloride, 10 mg/kg xylazine hydrochloride and 1.5 mg/kg Acepromazine. The tumor tissue required for implantation was obtained from VX-2 tumor bearing carrier rabbits. The carrier rabbits had VX-2 tumors implanted intramuscularly in the thigh. When the tumor reached approximately 1 cm in diameter (about 3 weeks from implantation), the tumor was excised, minced in sterile saline and VX-2 tumor fragments obtained. Two fragments were injected using a large bore needle into the thigh muscles of both legs in a recipient rabbit for tumor propagation. For experimental animals, after laparotomy, a fragment of tumor tissue (1 mm^3^) was implanted beneath the kidney capsule of the recipient rabbit.

The current experiment comprised 28 animals (7 in each of 4 groups). Fourteen days after tumor implantation the initial tumor volume was assessed based on serial (2.2 mm interval) T1 weighted axial MRI images (1.5 Tesla, GE Genesis-Signa) obtained 3 minutes following IV injection of 3 ml of Gadolinium. Tumor volume was assessed from the area of the contrast enhancing lesion in each section. The animals were assigned randomly into 4 groups before treatment start:

1. TTFields treated group: TTFields were applied by using the NovoTTF-100A device (NovoCure LTD., Haifa, Israel). An optimal frequency of 150 kHz and intensity of 1–2 V/cm were used. TTFields were switched sequentially between two perpendicular field directions.

2. Control group: sham electrode heated to mimic heat generated by the TTFields treatment. (38–39.9°C)

3. Paclitaxel (Medixel Injection., Taro Pharmaceutical Industries LTD., Israel) treated group: 5 mg/animal diluted in 100 ml of normal saline were infused intravenously over a period of 30 minutes. Premedication was given subcutaneous 8 hours before and immediately prior to Paclitaxel administration (Dexamathasone (Dexaveto-0.2 veterinary, V.M.D n.v/s.a Belgium) 0.5 mg/animal; Pramine (Metoclopramide HCL, Rafa Laboratories LTD., Israel) 1 mg/animal; Diphenhydramine (10%, Medical M., Israel) 10 mg/animal).

4. Combined TTFields and Paclitaxel treatment as above.

TTFields were delivered to awake and behaving rabbits through four insulated electrode arrays placed circumferentially around the animal's abdomen, caudal to the ribcage. The electrode insulation consisted of a high dielectric constant (>10,000) ceramic (PMN-PT) allowing efficient energy transfer through the insulation into the animals body at the given frequencies. The electrodes were connected by a spiral cable to a swivel mechanism at the top of the cage, enabling the free movement. TTFields were generated using the NovoTTF-100A system (NovoCure Ltd., Haifa, Israel). The animals were treated for 21 days continuously with MRI performed on days 14 and 21 for tumor volume assessment. The TTFields intensity within the kidneys of the rabbits, using this electrode configuration, is between 1–3 V/cm (based on both finite element mesh simulations and direct measurements using an invasive probe – data not shown).

### Pilot clinical trial

A single arm, pilot trial of the safety and efficacy of TTFields treatment was performed in 20 patients with histologically proven glioblastoma multiforme (GBM) that met the inclusion/exclusion criteria specified in Supplemental Material Appendix A (briefly, KPS 70–100%, Age ≥ 18). The trial was performed according to a protocol approved by the Na Homolce Institutional Review Board and the Czech Republic Ministry of Health. The patients were divided into two groups: The first group included 10 patients with recurrent GBM treated with TTFields alone following failure of maintenance Temozolomide [1]. The second group consisted of 10 newly diagnosed patients who were at least 4 weeks post radiation therapy, who received TTFields combined with maintenance Temozolomide. Prior to initiation of treatment, all patients underwent a baseline contrast MRI of the head, chest radiograph, EEG, ECG, complete blood & urine analyses, physical examination and neurological status. The patients were hospitalized for 1–3 days for observation and then released home where they received multiple 4-week courses of continuous NovoTTF-100A treatment until progression. The patients were seen once/month at an outpatient clinic where they underwent an examination similar to the initial one. TTFields were applied to the patients using the NovoTTF-100A device set to deliver 200 kHz, 0.7 V/cm (RMS) fields (at the center of the brain) in 2 perpendicular directions, 1 second in each direction sequentially. The TTFields were applied continuously using four insulated electrode arrays, each having a surface area of 22.5 cm^2^, placed on opposing sides of the head with the tumor positioned directly between the electrode pairs [1]. As previously reported, to avoid electrolysis at the electrode surface and intracellular ion concentration changes that accompany long term current application, the electrodes were completely insulated by a ceramic having a very high dielectric constant (>10,000) that allowed the generation of the necessary electric fields [1,2]. Using this electrode configuration, the lowest TTFields intensity at the center of the brain was 0.7 V/cm (RMS). This intensity was calculated using finite element mesh simulations and verified by direct measurement in large animals and a human volunteer [1].

The outcome endpoints of the study included safety, overall survival (OS) and progression free survival (PFS). Assessment of tumor response was based on monthly MRIs according to the Macdonald criteria [12]. Median OS and PFS were determined using Kaplan Meier curves [13]. In the first group, PFS in NovoTTF-100A treated patients was compared to a matched group of concurrent control patients who received salvage chemotherapy at recurrence (n = 18). PFS in Temozolomide/NovoTTF-100A treated patients was compared to the PFS of a matched group of concurrent control patients (n = 32) who received Temozolomide alone (according to the protocol described by Stupp et al. [14]). OS in both groups was compared to matched historical control data with the same Karnofsky performance score (>60) and age [14].

## Results

### Breast cancer cell cultures

#### Dose – response of culture exposure to TTFields, paclitaxel, doxorubicin and cyclophosphamide, alone and in combination

**Figure 1:**
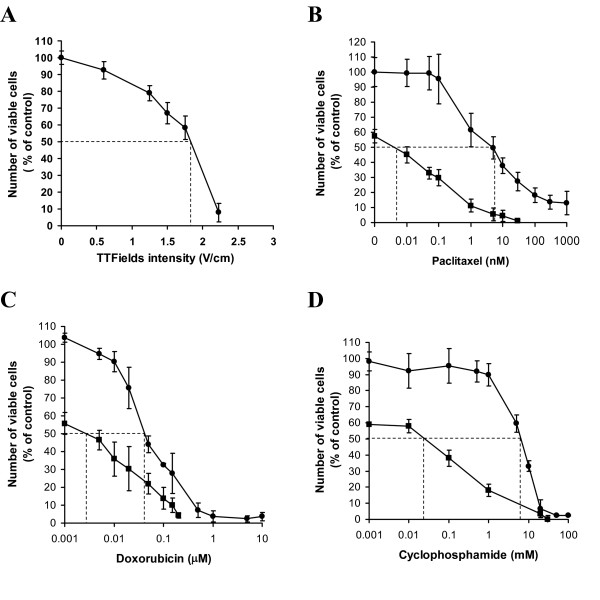
**Effect of 72 hour continuous application of TTFields and chemotherapeutic agents, separately and in combination on the cell proliferation of ER-negative MDA-MB-231 cells (presented as percent viable cells compared to control)**. (A) Percent viable cells vs. TTFields intensity. Effect of different concentrations of paclitaxel (B), doxorubicin (C) and cyclophosphamide (D), alone and in combination with TTFields of 1.75 V/cm. In B, C and D Filled Circles – represent drug alone; Filled Squares – drug in combination with TTFields. Each point represents mean values ± SEM of 18 to 36 replicate measurements. Dotted lines demarcate the IC_50 _values for each curve.

The relationship between TTFields intensity, at 150 kHz, and cell proliferation rate is given in Figure 1A. At the lowest field intensity of 0.63 V/cm there is no significant change in cell proliferation. For TTFields intensities of 1.25, 1.75 and 2.95 V/cm cell proliferation decreases (control = 100%) to: 90 ± 3%, 74 ± 4% and 25 ± 5%, respectively. The dose-response curves of cells exposed to paclitaxel, doxorubicin and cyclophosphamide, alone and in combination with 1.75 V/cm TTFields for 72 hours, are given in Figures 1B, C & D. For each drug alone there is a decrease in cell proliferation with increase in concentration. For cyclophosphamide and doxorubicin complete inhibition of proliferation is achieved at high drug concentrations. For paclitaxel, the inhibitory effect of the drug saturates at about 300 nM, near the 13% level, indicating that a fraction of the cells are insensitive to the agent. Combined treatment with TTFields and each of the chemotherapeutic agents caused a leftward shift of the dose response curves. This shift can be expressed as a decrease in the drug concentration leading to 50% inhibition of cell proliferation (IC_50 _– Table 1).

**Table 1:** IC_50 _for chemotherapeutic drugs alone and in combination with 1.75 V/cm TTFields after 72 hours of continuous treatment.

**Chemotherapy**	**IC_50 _(drug alone)**	**IC_50 _(drug-TTFields combination)**
Paclitaxel	5.00 nM	0.005 nM
Doxorubicin	0.04 μM	0.002 μM
Cyclophosphamide	6.60 mM	0.044 mM

#### Time course of the effects TTFields, paclitaxel, doxorubicin and cyclophosphamide

**Figure 2:**
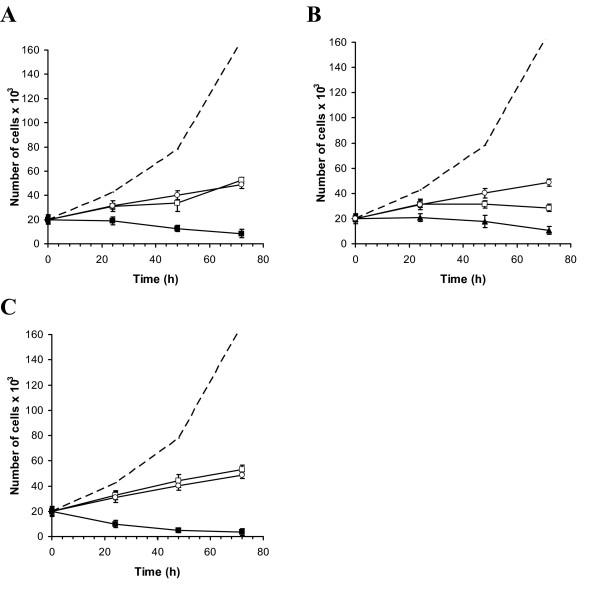
**Time course of the effects of 72 hour exposure of MDA cells to Paclitaxel (A), Doxorubicin (B) and Cyclophosphamide (C) alone and in combination with 1.75 V/cm TTFields**. Each graph shows the number of viable cells in culture over time in control cells (interrupted lines), drug alone (open squares), TTFields alone (open circles) and drug-TTFields combination (closed squares). Data are presented as mean ± SEM. Each experimental condition included 18–36 samples.

Figure 2 displays the time course of proliferation inhibition during a continuous 72 hour exposure to TTFields, paclitaxel, doxorubicin and cyclophosphamide alone and in combination with 1.75 V/cm TTFields. It is seen that in all cases the inhibition during combined exposure is greater than for the chemotherapeutic agent alone. The differences between the separate and combined effects increase with time.

#### Recovery from treatment

**Figure 3:**
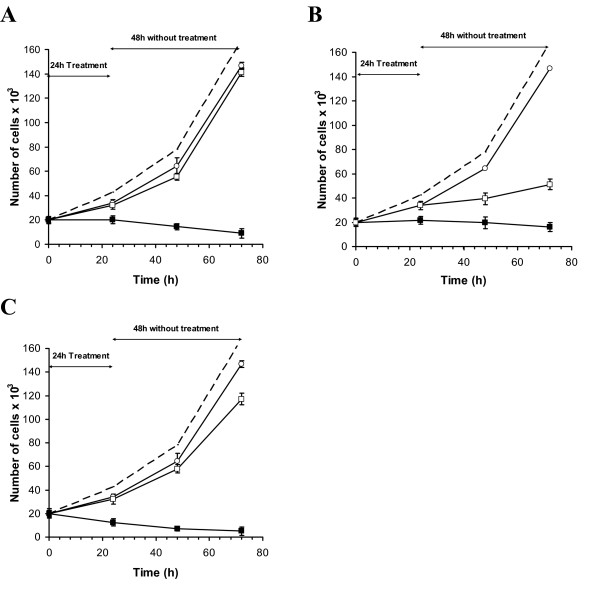
**Time course of recovery from 24 hour exposure to Paclitaxel (A), Doxorubicin (B) and Cyclophosphamide (C) alone and in combination with 1.75 V/cm TTFields**. Each graph shows the number of viable cells in culture over time in control cells (interrupted lines), drug alone (open squares), TTFields alone (open circles) and drug-TTFields combination (closed squares). Data are presented as mean ± SEM. Each experimental condition included 18–36 samples.

Figure 3 demonstrates that a 24 hour exposure to individual chemotherapeutic agents induces a reduction of approximately 25% in viable cell number compared to controls. The proliferation rate (slope of the graph) recovers almost completely during the following 48 hours, except for doxorubicin, where recovery is slower and delayed by about 24 hours. In contrast, addition of TTFields to any one of these chemotherapeutic agents results in irreversible and complete inhibition of cell proliferation rate manifested as a decrease in the number of cells in culture. For Cyclophosphamide there is an almost complete loss of viable cells after 72 hours of combined treatment.

### Glioma cell cultures

#### Combined effect of DTIC and TTFields in human glioma cell cultures

**Figure 4:**
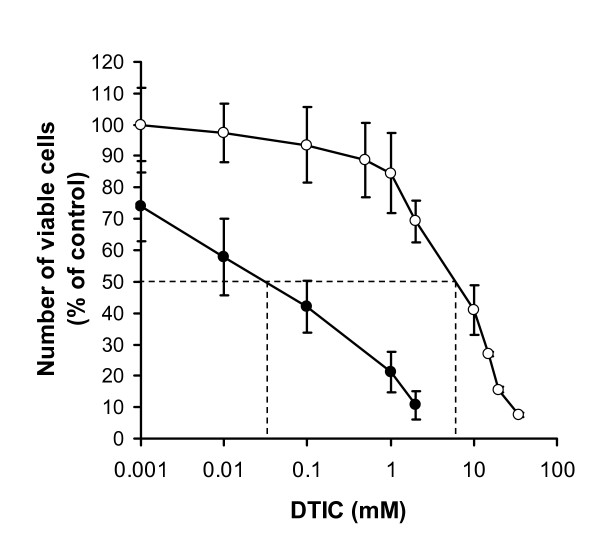
**Effect of light activated DTIC and TTFields (1.75 V/cm) on cell proliferation of U-118 glioma cells, presented as percent of viable cells compared to control**. Open Circles – 72 hours of DTIC treatment alone. Filled Circles – 72 h of Combined DTIC – TTFields treatment.

In order to asses the combination between Temozolomide and TTFields in glioma cells, DTIC and TTFields were applied alone and in combination to U-118 cells in culture. Both DTIC and Temozolomide act through a common degradation product (MTIC). Thus light activated DTIC was used for these experiments as described previously [15,16]. Figure 4 compares the DTIC dose-response curve, with that obtained with DTIC – TTFields combination. As we have shown in breast cancer cultures, the addition of TTFields to a chemotherapeutic agent causes a leftward shift in the dose-response curve in glioma cells as well. The IC_50 _for DTIC alone in Figure 4 is 6.4 mM, whereas the IC_50 _for combined DTIC-TTFields is two orders of magnitude lower (0.023 mM).

### Analysis of combination efficacy and sensitivity in-vitro

#### Combination indexes

The mode of interaction between TTFields and chemotherapeutic agents (synergism, additivity or antagonism) can be analyzed using Combination Indexes (CI) as described by [10,17]. In order to calculate the CIs for TTFields-Chemotherapeutic agents, the extent of inhibition of cell growth was assessed after 24 hours of treatment with Paclitaxel, Doxorubicin and Cyclophosphamide alone or in combination with different intensities of TTFields (0.625–1.75 V/cm; see Materials and Methods). Table 2 demonstrates that for breast cancer cells the CI for Doxorubicin is very close to 1, indicating additivity [10,11]. In contrast, for TTFields with Paclitaxel and Cyclophosphamide the CIs are <1 indicating additivity with a tendency towards synergism.

**Table 2:** Calculated Combination Indexes for human breast cancer (MDA-MB-231) cells treated with paclitaxel, doxorubicin or cyclophosphamide in combination with TTFields.

	Combination index		
	
	MDA-MB-231 cells		
	
TTFields intensity (V/cm)	Paclitaxel	Doxorubicin	Cyclophosphamide
	
	CI_40_	CI_50_	CI_50_
0.625	-	-	0.74
1.25	0.97	0.99	0.84
1.75	0.86	0.98	0.95

#### Dose reduction indexes

In order to assess the extent of possible chemotherapeutic dose reduction when applied in combination with TTFields, dose reduction indexes (DRI) for each drug-TTFields combination were calculated based on the methodology described by [11]. The DRIs for TTFields-drug interaction after 72 hours of combined treatment was 1316 for paclitaxel, 23 for doxorubicin, 152 for cyclophosphamide and 175 for DTIC (in U-118 glioma cells). Thus a significantly reduced dose (1–3 orders of magnitude lower drug concentration) may be used in combination with TTFields to achieve the same level of efficacy.

### Effect of combined paclitaxel and TTFields on VX2 tumors in rabbits

Prior to testing the combined efficacy of paclitaxel and TTFields on VX2 tumors implanted within the kidneys of rabbits, the dose-response of paclitaxel in this animal tumor model was determined. A dose of Paclitaxel leading consistently to a 15–20% inhibition in tumor growth (5 mg/rabbit) was chosen for subsequent combination experiments with TTFields.

**Figure 5:**
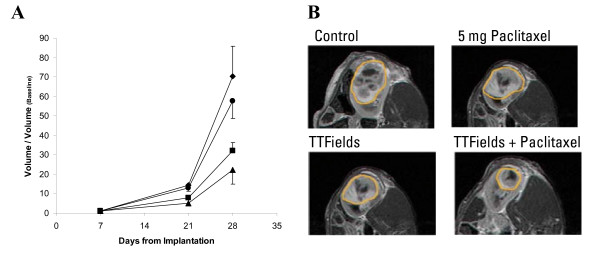
**Effect of combined Paclitaxel/TTFields on VX2 tumors in Rabbits**. A VX-2 Kidney tumor volumes were normalized to pre-treatment tumor volume (day 7) and are presented over time for; control (diamonds), 5 mg Paclitaxel (circles), TTFields (squares) and combined TTFields-Paclitaxel (triangles). The effect of combined TTFields and Paclitaxel is equal to the sum of the effects of either treatment alone at both time points measured during the study (2 and 3 weeks from treatment start; n = 23; bars are standard errors of means). B Exemplary MRIs of the maximal contrast enhancing tumor area (demarcated by orange boarders) in the kidneys of rabbits in each of the experimental groups (sham control, Paclitaxel 5 mg, TTFields 2 V/cm, combined Paclitaxel and TTFields).

As seen in Figure 5, untreated tumors increased in volume by a factor of 70 from baseline, Paclitaxel treated tumors grew by a factor of 58 from baseline, TTFields treated tumors grew by a factor of 34 from baseline and tumors treated by TTFields-Paclitaxel combination grew by a factor of 22 from baseline. Thus the TTFields-Paclitaxel combination treatment inhibited tumor growth by 69% compared to the growth of control tumors, while Paclitaxel alone inhibited tumor growth by 15% compared to the growth of control tumors, and TTFields alone by 53% compared to the growth of control tumors. Thus, additivity was seen between TTFields and Paclitaxel at the intensity and concentration used. Differences between curves were statistically significant (p < 0.01; ANOVA).

### Pilot clinical trial in GBM patients

Twenty patients with histological diagnosis of GBM were treated continuously for an average of 1 year (range 2.5–24 months). Ten recurrent GBM patients were treated with TTFields alone as salvage therapy. Ten newly diagnosed GBM patients, that had undergone surgery and thereafter received radiation therapy with adjuvant Temozolomide, were treated with the combination of TTFields in parallel to maintenance Temozolomide [14]. In both groups of patients no device related serious adverse effects were observed. The only device related toxicity reported was a dermatitis which appeared most often (18 of 20 patients) during the second month of treatment. The severity of the dermatitis decreased upon use of topical corticosteroids and periodic electrode relocation. The dermatitis continued for the duration of treatment and resolved completely within days to weeks from treatment termination.

In the second group, no increase in Temozolomide related adverse events was seen due to the combination with TTFields (see Table 3).

**Table 3:** Toxicities by grade and causality in the newly diagnosed GBM patients treated with combined TTFields-Temozolomide.

	**Grade**		**Causality assessment**
	**I-II**	**III-IV**	

Elevated LFTs	6/10	0/10	Anti Epileptic Drugs
Hyperglycemia	4/10	0/10	Oral Steroids
Anemia	6/10	0/10	Temozolomide
Thrombocytopenia	2/10	0/10	Temozolomide
Leucopenia	3/10	0/10	Temozolomide
Headache	2/10	0/10	Underlying disease
Seizures	1/10	0/10	Underlying disease
Dermatitis	10/10	0/10	NovoTTF-100A

**Figure 6:**
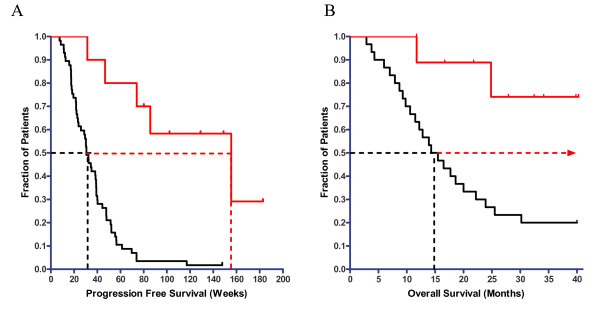
**Kaplan Meier curves for A – progression free survival (PFS) and B – overall survival (OS) of newly diagnosed GBM patients receiving either combined TTFields – Temozolomide treatment or Temozolomide treatment alone**. Red line – patients receiving combined TTFields – Temozolomide treatment (n = 10). Black line – concurrent/historical control patients that received Temozolomide treatment alone. A – The difference between the PFS curves is highly significant – Log-Rank Test (P = 0.0002), Hazard Ratio 3.32 (95%CI 1.9–5.9). B – The difference between the OS curves is highly significant – (Log-Rank Test; P = 0.0018). Dashed lines mark the median values for each curve.

As reported previously [1], both progression free survival (PFS) and overall survival (OS) in the recurrent GBM salvage therapy group were at least double that of concurrent and historical controls, respectively. The efficacy of the TTFields-Temozolomide combination in the second group of patients was assessed using Kaplan Meier curves [13] of PFS and OS. The Kaplan Meier curves for the PFS of these patients, treated by combined TTFields – Temozolomide are shown in Figure 6A. The median PFS of the combination treated patients is 155 weeks versus 31 weeks for concurrent controls treated with maintenance Temozolomide alone. Note that 5 of 10 patients are currently progression free. Figure 6B compares the OS of the patients that received the combination treatment (Red line) with a matched historical control (KPS>60, Median age 54) (Black line [14]). It is seen that for the TTFields – Temozolomide combination treated patients, the Median OS > 39 months versus about 14.7 months for matched historical control patients who received maintenance Temozolomide alone. It should be noted that at the time of this report 8 of 10 patients, receiving the TTFields-Temozolomide combination treatment, are alive.

## Discussion

Cancer treatment with drug combinations was introduced in order to improve therapeutic indexes through dose reduction of each drug and increase treatment efficacy. In this study the exposure of cancer cells to combined chemotherapy and TTFields was studied in cell cultures, an animal tumor model and in a pilot clinical trial in recurrent and newly diagnosed GBM patients. The results of this study support the possibility that TTFields may be used, not only as an effective stand alone anti-proliferation agent (as shown previously in [1]), but also as an effective adjuvant that enhances chemotherapy efficacy without an increase in toxicity. In addition to this increase in efficacy, these results raise the possibility of dose reduction of chemotherapy when used in combination with TTFields. This is of outmost importance since, at tolerable doses the efficacy of available cancer therapeutic agents is often far from optimum while being associated with a high degree of toxicity.

**Figure 7:**
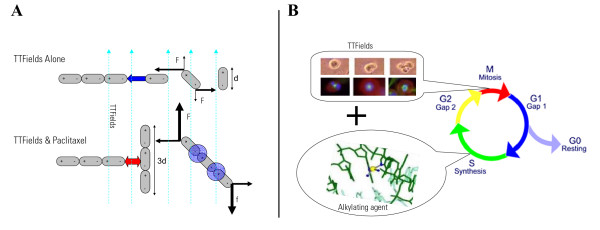
**Mechanisms of potentiation of chemotherapeutic efficacy by TTFields**. A Tubulin chains are elongated by Paclitaxel, leading to an increase in the average dipole moment of free tubulin chains (d – length of an individual tubulin dimmer; f – force between the microtubule chain and the dimmer; F-force acting on the tubulin dimmers by TTFields; Arrow length is proportional to the intensity of these forces). The forces TTFields exert on these larger dipoles, F, are enhanced leading to an increase in the disruption of the mitotic spindle by TTFields. B TTFields act as an M-phase inhibitor, while alkylating agents act at the G and S phases of the cell cycle. This separation between cell cycle phases affected explains the additivity seen experimentally.

With regards to the mechanisms involved, one may assume that tumor cells are sensitized to TTFields by chemotherapy, much like another well established physical therapy – ionizing radiation [8,18,19]. In the specific case of Paclitaxel, one of the most commonly used treatments for late-stage human breast cancer [20], the combined effect may be attributed to their similar site of action – the spindle microtubules [1,2,21]. Taxanes act by stabilizing the link between individual tubulin dimmers [21]. As illustrated schematically in Figure 7A taxanes increase the length of tubulin filaments within the cell. One of the mechanisms of action of TTFields is the misalignment of mitotic spindle filaments as a result of TTFields forces on tubulin chains [2]. The increase in filament length due to taxanes, increases the dipole moment of these macromolecules, leading to an increase in the TTFields induced forces and thus to a higher sensitivity of the cell to TTFields (see Figure 7A).

Doxorubicin that has a broad spectrum of activity both in experimental tumor models and in human malignancy, affects both DNA and RNA synthesis [22]. Cyclophosphamide (an alkylating agent) inhibits DNA replication by interfering with the separation of the double stranded DNA essential for transcription [23]. As illustrated in Figure 7B, since TTFields act at a completely different stage (M phase) of the cell cycle from both these agents, additivity between chemotherapy and TTFields can be expected.

Since the data for newly diagnosed GBM patients, which points to well over a 300% increase in PFS and OS, was obtained only with combination treatment, one cannot directly separate the TTFields effects from the chemotherapeutic effect. However, if we assume that the TTFields therapeutic efficacy for newly diagnosed patients is similar to recurrent GBM, i.e. the median of OS is increased by 270% [1] while the published Temozolomide data indicates an increase of about 20% in OS compared to ionizing radiation treatment alone [14], the results presented in Figure 6 point towards additivity between TTFields and Temozolomide. It is important to note that this significant increase in efficacy was obtained without any increase in device or drug related toxicity (see table 3).

An additional important finding is that both 24 h and 72 h combination treatments in-vitro result in severe irreversible cellular damage in contrast to chemotherapy alone. This result strengthens the assumption that combination therapy with TTFields may be much more effective than treatment by individual agents.

## Conclusion

The results of the present study support the notion that TTFields may be used clinically not only as an anti-proliferation agent as shown before [1], but also as effective sensitizers of currently used chemotherapeutic agents. Such sensitization was not shown to be associated with any additional systemic toxicity. Moreover, as demonstrated by the high DRIs calculated in this study, chemo/TTFields combinations are expected to provide the same or even greater therapeutic efficacy with much lower drug concentrations thus lowering further the overall toxicity.

## Competing interests

EK, RSS, AI, DM, ZG, ES and YW are employees of NovoCure Ltd.

YP has a minority holding in NovoCure Ltd.

VD, FT, JV and DG have no competing interests.

## Authors' contributions

EK – planned the pre-clinical and clinical experiments, supervised their execution, analyzed results and wrote parts of the manuscript. RSS and ET – Performed the in-vitro experiment and assisted in the in-vivo experiments. DM, ZG and AI – Performed the in-vivo experiments. DG – Performed the MRI imaging for the in-vivo experiments. YW – Planned the medical devices and treatment parameters for all experiments. VD, FT and JV – performed the clinical trial in GBM patients (clinical investigators). YP – invented the concept of TTFields, helped interpret all results and wrote the majority of the manuscript.

## Appendix

Appendix A – Eligibility criteria for the pilot GBM trial

Inclusion criteria:

Histologically proven diagnosis of GBM.

Age over 18 years.

Karnofsky scale ≥ 70.

Participants of child bearing age had to be receiving efficient contraception.

Willing and able to sign an informed consent prior to participation in the study.

Exclusion criteria:

Patients actively participating in another clinical trial

Patients who received any anti-tumor therapy in the four weeks prior to trial initiation (steroids are permitted; however, the dose must be stable or decreasing during the trial).

Patients suspected of suffering from radiation necrosis (according to a PET scan).

Pregnancy

Patients with one of the following co-morbidities:

Patients with an implanted pacemaker or documented arrhythmias.

Significant renal, hepatic or hematologic disease.

Significant additional neurological disorder:

Seizure disorder unrelated to the patient's tumor

Pre-existing dementia

Progressive degenerative neurological disorder

Meningitis or encephalitis

Hydrocephalus associated with increased intracranial pressure (ICP)

## Pre-publication history

The pre-publication history for this paper can be accessed here:


